# Commentary: Differential associations between obesity and behavioral measures of impulsivity

**DOI:** 10.3389/fpsyg.2016.00949

**Published:** 2016-06-21

**Authors:** Casey K. Gardiner, Hollis C. Karoly, Angela D. Bryan

**Affiliations:** Department of Psychology and Neuroscience, University of Colorado BoulderBoulder, CO, USA

**Keywords:** obesity, reward, reward processing, delay discounting, inhibition (psychology), response inhibition

Growing evidence supports the concept of a “cognitive profile” (Jansen et al., 2015) of obesity, a set of interrelated cognitive patterns, largely influenced by executive functioning, that may underlie obesogenic behaviors, especially in contemporary environments, which are saturated with food cues. These cognitive deficits include attentional biases toward food (Castellanos et al., 2009; Yokum et al., 2011) and discounting of future rewards (Weller et al., 2008). A greater understanding of these cognitive patterns may aid the development of more effective behavioral interventions for obesity, whether through training to minimize cognitive biases, designing interventions to sidestep the effect that these cognitive patterns may have on behavior, or even harnessing biases to drive more adaptive behaviors.

In order to more completely understand the relationships between cognition, behavior, and obesity, it is necessary to identify the specific constructs that are most strongly related to weight status. To this end, Lawyer et al. (2015) sought to disentangle the differential associations between weight status and three aspects of impulsivity (a key component of executive function): delay discounting, probability discounting, and response inhibition. They propose that their results demonstrate that the former two constructs are related to body mass index and obesity, while the latter is not. We would like to propose that this conclusion regarding inhibition warrants further careful examination.

Specifically, the Lawyer et al. study employed a standard version of the Stop Signal Reaction Task (SSRT), which may not be the best measure of inhibition for these purposes. First, while the two discounting tasks in the study necessarily involved reward-related cognition (i.e., monetary choices), the SSRT assessed inhibition in a task that lacked any motivational salience (i.e., pressing or not pressing a button, depending on what letter was displayed on the screen). Evidence from cognitive psychology and neuroscience suggests that motivational factors can play a critical role in performance on cognitive control tasks (Botvinick and Braver, 2015), including those that test response inhibition (Leotti and Wager, 2010). Thus, it is plausible that the strength of the relationships between weight status and discounting appear greater than that between weight and inhibition due to the motivational nature of the discounting tasks, rather than the difference between discounting and inhibition *per se*. Lawyer and colleagues' results may thus provide stronger evidence for the importance of reward processing in obesity and obesogenic behaviors.

Importantly, neural and physiological reward signaling are crucial contributors to both hedonic and homeostatic eating behaviors (Murray et al., 2014), and a growing literature documents that reward processing is implicated in both eating behaviors and weight status. Neural dopamine signaling is a crucial mechanism of disinhibited eating in rodent models (Johnson and Kenny, 2010), and in humans, it is also clearly involved in eating behaviors and weight status (Volkow et al., 2011). Notably, polymorphisms in dopamine receptor genes are associated with food reinforcement, energy intake, and weight gain (Epstein et al., 2007b; Stice et al., 2010). Additionally, when compared to lean individuals, those who are obese (Stoeckel et al., 2008) or obesity-prone (Cornier et al., 2013) display differential activity in a variety of brain regions implicated in reward processing when viewing or consuming food. In addition, neural reward responses to food images can prospectively predict weight gain (Demos et al., 2012).

Moreover, reward processing discrepancies extend to non-food rewards (Wu et al., 2016). Obese individuals' brains respond differently than do their healthy weight counterparts, with greater activation in reward-relevant regions (e.g., striatum), when anticipating (Balodis et al., 2013) and receiving (Opel et al., 2015) monetary rewards, and brain activity during monetary choices can predict weight change in obese individuals (Kishinevsky et al., 2012). Further, behavioral reward processing, including delay discounting (e.g., Weller et al., 2008) and food reinforcement (e.g., Saelens and Epstein, 1996; Epstein et al., 2007a), differs between obese and healthy-weight individuals, such that the former group of individuals tends to display greater propensities to discount the value of future rewards as well as to find food more reinforcing (or rewarding). Thus, as evidenced by this broad literature (including Lawyer et al.'s results regarding delay and probability discounting), reward processing is strongly implicated in eating behaviors, weight status, and weight change, and therefore its effects must be considered in examinations of cognition and obesity, *including when examining response inhibition.*

Though the specific SSRT task used by Lawyer et al. had no motivational component, it is possible to assess response inhibition in motivationally salient contexts, including those involving money (e.g., Demurie et al., 2013) or other appetitive cues. For example, our group adapted the standard SSRT neuroimaging paradigm to incorporate motivationally salient alcohol cues (images of alcohol and control beverages) that allow us to examine the interaction between impaired inhibitory control and reward processing in alcohol use disorders. We demonstrated that successful response inhibition during the motivationally salient alcohol cue trials (compared to control trials) was positively associated with activation in anterior cingulate, supplementary motor, and frontal inferior brain regions, and this activation was positively related to severity of alcohol use (Karoly et al., 2014). These findings indicate that compensatory error detection and inhibitory control resources may be recruited in the brains of individuals with severe alcohol problems during successful response inhibition in the context of motivationally salient alcohol cues; it is entirely plausible that similar effects exist for weight status and food cues.

Eating behavior researchers have employed behavioral and neural measures of response inhibition in tasks that include motivationally salient food images, with findings demonstrating that response inhibition in motivationally salient contexts is, in fact, associated with eating behaviors, weight status, and weight change (e.g., Batterink et al., 2010; Jasinska et al., 2012; Nederkoorn et al., 2012; Meule et al., 2014a,b; Veling et al., 2014; Allom and Mullan, 2015; Lawrence et al., 2015). Moreover, such tasks may be more relevant to understanding inhibition in real-world contexts, such as when individuals must choose whether or not to eat physiologically rewarding, energy-dense foods.

In sum, Lawyer et al. present an important step toward the identification of the cognitive factors associated with weight status and obesity. Their results clearly indicate that the propensities to discount delayed and probabilistic rewards are associated with weight status. However, their conclusion that response inhibition is unrelated to weight status requires further examination, given that prior research suggests that response inhibition is implicated in eating behaviors and weight status. Crucially, it appears that response inhibition, like discounting measures of impulsivity, is associated with weight status when it is assessed in a reward-related context. Outside of the laboratory, where individuals are inundated with opportunities to consume highly rewarding food stimuli, failures of response inhibition can underlie behaviors that contribute to the onset and maintenance of obesity. Thus, it is necessary for future research to continue to examine the nature of response inhibition among obese versus healthy-weight individuals in motivationally salient contexts. Reward processing is a crucial biobehavioral component of both cognitive performance and weight status, and thus its role cannot be ignored when examining their relationship.

## Author contributions

CG, HK, and AB conceived of the ideas, drafted the manuscript, reviewed the manuscript, and approve of the final version of the manuscript.

## Funding

CG and HK are supported by National Science Foundation Graduate Research Fellowships (US; DGE 1144083). The content is solely the responsibility of the authors and does not necessarily represent the official views of the National Science Foundation.

### Conflict of interest statement

The authors declare that the research was conducted in the absence of any commercial or financial relationships that could be construed as a potential conflict of interest.
